# Supplementary material for: The genome sequence of the Andalusian wall lizard, Podarcis vaucheri (Boulenger, 1905)
Source: Wellcome Open Res. 2025 May 20;10:265. [Version 1] doi: 10.12688/wellcomeopenres.24168.1 (PMC12743217; doi:10.12688/wellcomeopenres.24168.1)
Supplement: Peer review history

# Reviewer report: Reviewer response for version 1

**DOI:** 10.21956/wellcomeopenres.26659.r138095

**Published:** 2025-12-26

**Authors:** Jorge Gutiérrez-Rodríguez

**Affiliations:** 1 Universidad Nacional Autonoma de Mexico Instituto de Ecologia (Ringgold ID: 98857), Mexico City, Mexico City, Mexico

The manuscript describes a high-quality genome sequencing and assembly effort that employs cutting-edge methodologies, including PacBio HiFi long reads and Hi-C phasing. The clear documentation of procedures and the thorough reporting of quality metrics align well with the highest standards for contemporary genome projects (e.g., those of the EBP). The generation of a haplotype-resolved, chromosome-level assembly represents a notable scientific contribution.

 Minor comments:

 In the sentence “During curation, it was noted that The Z and W chromosomes were identified based on their single-copy status within a diploid assembly and synteny analysis with Podarcis raffonei (GCA_027172205.1)”, please change “The” to “the”.

 In the sentence: “P. vaucheri is one of three species in the Ibero-Maghrebian group of Podarcis for which a reference genome is constructed (the other two being P. bocagei and P. liolepis)” — add citations.

Are sufficient details of methods and materials provided to allow replication by others?

Yes

Is the rationale for creating the dataset(s) clearly described?

Yes

Are the datasets clearly presented in a useable and accessible format?

Yes

Are the protocols appropriate and is the work technically sound?

Yes

Reviewer Expertise:

Evolutionary Biology

I confirm that I have read this submission and believe that I have an appropriate level of expertise to confirm that it is of an acceptable scientific standard.

# Reviewer report: Reviewer response for version 1

**DOI:** 10.21956/wellcomeopenres.26659.r138098

**Published:** 2025-12-02

**Authors:** Hong-Xin Xie

**Affiliations:** 1 University of Glasgow, Glasgow, UK

The authors present a high-quality, haplotype-resolved, chromosome-level genome assembly of the Andalusian wall lizard *Podarcis vaucheri*, including both W and Z sex chromosomes. The Data Note is well written and provides sufficient biological context along with clear descriptions of the sequencing and assembly methods. The use of PacBio HiFi reads combined with Hi-C scaffolding is consistent with current best practices, and the resulting assembly appears to be of comparable quality to recently published squamate genomes. Overall, the manuscript meets the expectations for a genome Data Note and will be a valuable resource for the community. I have only two minor comments:

1. Figure 5 legend: The legend states that “Chromosomes are shown in order of size,” but the first two chromosomes depicted do not appear to be the largest; they seem to correspond to the sex chromosomes. It may be worth double-checking the chromosome ordering or clarifying the legend.
2. Assembly QV values: In the assembly quality metrics, the manuscript reports: “For haplotype 1, the estimated QV is 63.3, and for haplotype 2, 63.3. When the two haplotypes are combined, the assembly achieves an estimated QV of 63.3.” Given that all three values are identical, it may be useful to confirm whether this is correct or if a minor typo has occurred.

Are sufficient details of methods and materials provided to allow replication by others?

Yes

Is the rationale for creating the dataset(s) clearly described?

Yes

Are the datasets clearly presented in a useable and accessible format?

Yes

Are the protocols appropriate and is the work technically sound?

Yes

Reviewer Expertise:

genomics, evolution, herpetology

I confirm that I have read this submission and believe that I have an appropriate level of expertise to confirm that it is of an acceptable scientific standard.
